# Evaluating the Effects of a Rent Subsidy and Mentoring Intervention for Youth Transitioning Out of Homelessness: Protocol for a Mixed Methods, Community-Based Pilot Randomized Controlled Trial

**DOI:** 10.2196/15557

**Published:** 2019-12-20

**Authors:** Naomi S Thulien, Nicole Kozloff, Elizabeth McCay, Rosane Nisenbaum, Andrea Wang, Stephen W Hwang

**Affiliations:** 1 School of Nursing McMaster University Hamilton, ON Canada; 2 MAP Centre for Urban Health Solutions Li Ka Shing Knowledge Institute of St Michael's Hospital Toronto, ON Canada; 3 Department of Psychiatry University of Toronto Toronto, ON Canada; 4 Slaight Family Centre for Youth in Transition Centre for Addiction and Mental Health Toronto, ON Canada; 5 Daphne Cockwell School of Nursing Ryerson University Toronto, ON Canada; 6 Applied Health Research Centre Li Ka Shing Knowledge Institute of St Michael's Hospital Toronto, ON Canada; 7 Department of Health Research Methods, Evidence, and Impact McMaster University Hamilton, ON Canada; 8 Division of General Internal Medicine, Department of Medicine University of Toronto Toronto, ON Canada; 9 Dalla Lana School of Public Health University of Toronto Toronto, ON Canada

**Keywords:** homeless youth, community integration, qualitative research, randomized controlled trial, housing, mentorship

## Abstract

**Background:**

Although the risk factors associated with young people entering and becoming entrenched in street life have been thoroughly investigated, peer-reviewed evidence is scarce to nonexistent for rigorous interventions targeting social integration outcomes for young people who have experienced homelessness. From the limited research that has been done, emerging evidence signals that, although structural supports such as subsidized housing and social service providers are important, these resources alone are insufficient to help young people integrate into the mainstream society.

**Objective:**

The overarching aim of this study is to assess whether and how rent subsidies and mentorship influence social integration outcomes for formerly homeless young people living in market rent housing in 3 Canadian cities. The primary outcome measures for this study are community integration (psychological and physical) and self-esteem at 18 months. Secondary outcomes include social connectedness, hope, and academic and vocational participation at 18 months. Exploratory outcomes include income, perceived housing quality, engulfment, psychiatric symptoms, and participant perspectives of intervention barriers and facilitators.

**Methods:**

This is a convergent mixed methods, open-label, 2-arm parallel randomized controlled trial (RCT) with 1:1 allocation embedded within a community-based participatory action research (CBPAR) framework. The intervention will provide 24 young people (aged 16-26 years), who have transitioned out of homelessness and into market rent housing within the past year, with rent subsidies for 24 months. Half of the young people will also be randomly assigned an adult mentor who has been recruited and screened by 1 of our 3 community partners. Data collection will occur every 6 months, and participants will be followed for 30 months.

**Results:**

Ethical approval for this study has been obtained from the Providence, St Joseph’s, and St Michael’s Healthcare Research Ethics Board (number 18-251). Enrollment took place from April 2019 to September 2019. Preliminary analysis of the baseline quantitative and qualitative data is underway.

**Conclusions:**

This pilot RCT will be the first to test the impact of economic and social support on meaningful social integration for formerly homeless young people living in market rent housing. We believe that the mixed methods design will illuminate important contextual factors that must be considered if the intervention is to be scaled up and replicated elsewhere. Importantly, the CBPAR framework will incorporate the perspectives of the community, including formerly homeless young people, who are in the best position to determine what might work best in the context of their lives.

**Trial Registration:**

Clinicaltrials.gov NCT03779204; https://clinicaltrials.gov/ct2/show/NCT03779204.

**International Registered Report Identifier (IRRID):**

DERR1-10.2196/15557

## Introduction

### Background and Rationale

Young people comprise almost 20% of the homeless population in Canada [1]. An estimated 35,000 to 40,000 Canadian youth (aged 13-25 years) are homeless at some point during the year and at least 6000 on any given night [2,3]. Although the risk factors associated with young people entering and becoming entrenched in street life have been thoroughly investigated, peer-reviewed evidence is scarce to nonexistent for rigorous interventions targeting housing outcomes, life trajectories, quality of life, and social integration for young people who have experienced homelessness [4-7]. It must be noted that the concept of social integration is complex and often inconsistently defined and poorly measured [8]. For the purpose of this study, we drew from the literature on the social determinants of health and social exclusion and adopted a holistic definition of social integration, incorporating both the tangible (eg, access to education and a living wage) and intangible (eg, sense of connection and belonging) aspects of meaningful and equitable societal participation [4,9,10].

Intuitively, it may seem that one important way to improve the life trajectories of young people experiencing homelessness is to provide them with a home. However, from the limited research that has been done in this area, we know that formerly homeless young people continue to experience significant challenges—particularly when it comes to mainstream social integration—even after they are "successfully" housed [11,12]. Moreover, these challenges seem to persist regardless of the type of housing (eg, subsidized vs market rent) provided [13-15].

#### Housing and Social Integration

A longitudinal mixed methods study with 51 formerly homeless young people (aged 16-25 years) living in 2 major urban centers in Canada found that, despite living in stable or semistable accommodations (53% lived in subsidized housing), participants continued to face substantial social integration challenges such as poverty-level incomes and limited mainstream social networks [14,16,17]. Over the course of 1 year, these challenges contributed to a significant decline in hope, no gains in community integration, and a sense of being *stuck* [14,16,17]. Notably, these challenges were significantly worse for participants paying market rent [14].

A subgroup analysis of 156 young people (aged 18-24 years) with mental health challenges who participated in a 24-month randomized controlled trial (RCT) of *Housing First* (access to subsidized housing and comprehensive social service support) in 5 Canadian cities—the largest RCT of Housing First to date—indicates similar findings of social integration challenges despite the provision of a home [15]. Although the young people who received the Housing First intervention achieved significantly better housing stability compared with the treatment-as-usual group, they did not experience improvements to other outcomes such as employment, generic quality of life, and community integration relative to the treatment-as-usual group [15].

In line with the findings from both these studies, a 10-month ethnographic study conducted by the members of our team with 9 formerly homeless young people (aged 18-24 years) living in Canada’s largest city highlighted that, despite the appearance of housing stability in market rent housing, the young people were experiencing significant social integration challenges [11,12]. Study participants described living a precarious existence, attributed in part to the chronic stress and exhaustion of living in poverty and to their limited knowledge about how to move forward in life [11,12]. In addition, we noted that the participants underutilized transition-related social support (eg, food banks and employment counseling) because these types of support tended to be deficit-focused (eg, focused on what youth did not have and not on what they had achieved) and located in areas (eg, homeless shelters) that reminded them of their old identities as homeless youth. Identities primarily defined, or in other words *engulfed*, by homelessness may in fact preclude young people from achieving meaningful social integration [11].

#### Mentorship and Social Integration

As previously mentioned, little evidence exists for effective interventions that target social integration outcomes for young people who have experienced homelessness. This includes evidence on the impact of mentorship. In fact, for formal mentorship programs in general, meta-analyses have only found small overall positive effect sizes on the psychological, emotional, behavioral, and educational functioning of participating young people [18,19]. However, there is some emerging evidence on the benefits of *natural mentors*—generally defined as an important, encouraging, nonparental adult who exists in a youth’s social network—that may be transferable to youth who have experienced homelessness.

A systematic review of natural mentoring for youth (aged 13-25 years) transitioning out of foster care showed that the young people benefited from a supportive adult not “tasked with enforcing daily rules and addressing misbehavior” and that this sort of intervention resulted in improved behavioral, psychosocial, and academic outcomes [18]. A meta-analysis of natural mentoring in youth (aged 13-24 years) discovered similar positive outcomes in the domains of social and emotional development and academic and vocational functioning [19]. Furthermore, the risk status of youth (eg, young people experiencing homelessness or living in foster care) did not moderate these positive outcomes [19]. A small (n=23) qualitative study of natural mentoring relationships among young people (aged 14-21 years) experiencing homelessness also supported these positive findings, and the authors suggested that “natural mentors could feasibly serve as a bridge in a coordinated effort to assist youth out of homelessness” [20]. Taken together, these studies show promise for mentoring interventions that incorporate the positive characteristics of natural mentors (ie, more of a friendship-like *coach* or *cheerleader* role) for young people who have experienced homelessness. Moreover, although traditional natural mentoring relationships tend to emerge organically, they can be facilitated and supported programmatically as well [15].

From the limited research that has been done with young people transitioning away from homelessness, the emerging evidence signals that, although structural supports such as subsidized housing and social service providers are important, these resources alone are insufficient to help young people integrate into the mainstream society. As it currently stands, the burden for achieving meaningful social integration is on young people, who continue to be marginalized despite achieving stable or semistable housing. Connecting formerly homeless youth with an adult who exhibits the relationship-based components of natural mentoring that young people value most (eg, genuine interest in their well-being and belief in their ability to succeed) may be key to helping young people move forward and integrate into the mainstream.

### Objectives

The overarching aim of this mixed methods study is to assess whether and how rent subsidies and mentorship influence social integration outcomes for formerly homeless young people living in market rent housing in 3 urban settings.

#### Quantitative Objectives

The primary objective is to determine whether rent subsidies and mentorship result in better social integration outcomes than only receiving rent subsidies with regard to (1) community integration (psychological and physical) and (2) self-esteem.

The secondary objectives are to determine whether rent subsidies and mentorship result in better social integration outcomes than only receiving rent subsidies with regard to (1) social connectedness, (2) hope, and (3) sustained academic and vocational participation.

The exploratory objectives are to assess whether rent subsidies and mentorship result in better social integration outcomes than only receiving rent subsidies with regard to (1) income, (2) perceived housing quality, (3) sense of engulfment, and (4) psychiatric symptoms.

#### Qualitative Objectives

The qualitative objectives of the study are (1) to explore what the study participants (young people and mentors) found most beneficial about the intervention and how it could be improved and (2) to facilitate a more comprehensive and contextualized understanding of the quantitative data.

## Methods

### Design and Setting

This is a convergent mixed methods (ie, quantitative and qualitative data are collected concurrently and the findings, combined), open-label, 2-arm parallel RCT with 1:1 allocation embedded within a community-based participatory action research (CBPAR) framework [21,22]. We believe a mixed methods RCT is appropriate given the complex explanatory pathways (ie, social and behavioral processes that may act independently and interdependently) of this intervention [23]. The study will be conducted in 3 Canadian cities in Ontario: Toronto (population 2.8 million), Hamilton (population 552,000), and St Catharines (population 133,000).

### Community-Based Participatory Action Research Methodology

With the goal of reducing health inequities through knowledge and action, CBPAR can be a powerful tool for those working with marginalized populations [24-27]. Our study team is committed to drawing on the following key principles of CBPAR from study inception to dissemination [24-27]:

- Research participants are viewed as experts in their own lives.
- Concerted effort to reduce power imbalances between the researchers and the community.
- Equal value placed on academic knowledge and experiential knowledge.
- Commitment to producing practical, actionable data to build community capacity and improve the lives of the research participants.
- Duty to remain invested with the community beyond the life of the research project.

### Eligibility Criteria and Recruitment

A total of 24 young people aged between 16 and 26 years, who have experienced homelessness within the past year and are living in market rent housing, will be collaboratively recruited by our research team and our 3 community partners: Covenant House Toronto, Living Rock Ministries (Hamilton), and the Raft (St Catharines). We adopted the Canadian Observatory on Homelessness’ definition of homelessness and defined homelessness to include young people who are unsheltered (eg, sleeping on the streets), emergency sheltered (eg, homeless shelter), or provisionally accommodated (eg, time-limited subsidized housing) [28].

In addition to the above age and housing inclusion criteria, study participants must be able to provide free and informed consent, fluent in English, planning on staying in or nearby the community in which they were recruited for the duration of the 24-month study, and willing to be matched with an adult mentor who has been screened and recommended by one of our 3 community partners.

Young people will be excluded from the study if they are in imminent danger of losing their housing (eg, facing jail time or impending eviction) and are enrolled in another study with enhanced financial and social support.

Potential participants will be screened for eligibility over the phone by the study coordinator. If a participant meets the eligibility criteria, the study coordinator will arrange a face-to-face meeting between the young person and a member of the research team. During this initial meeting, informed consent will be obtained and the youth will be enrolled in the study. Enrolled participants will be asked to provide baseline quantitative data before being randomized into the intervention (rent subsidies plus mentoring) or the active comparator (rent subsidies only) group.

### Sample Size

This pilot study was designed with the intention of generating data and hypotheses for a full-scale study. The sample size was chosen based on the financial resources available to provide substantial rent supplements over a 2-year period, and no formal sample size calculation was performed [29].

### Randomization

Participants at each of the 3 study sites (Toronto [n=12], Hamilton [n=6], and St Catharines [n=6]) will be randomized using block randomization. Randomization will be balanced by site based on random block sizes of 2 and 4. The advantage of using block randomization is to uniformly distribute participants into treatment groups within each site [30]. As small block sizes may increase the risk of guessing the allocation procedure and subsequently introducing bias into the enrollment procedure, random block sizes will be used to avoid this potential selection bias [31].

The study biostatistician will generate a unique randomization schedule for each site using SAS software (SAS Institute Inc). A research coordinator at St Michael’s Hospital, who is not affiliated with the study, will be the only person with access to the randomization schedule. She will prepare sealed and sequentially numbered envelopes, separated by site (Toronto, Hamilton, and St Catharines). After each participant has been enrolled and has participated in baseline data collection, a member of the study team will select a randomization envelope from the sequentially ordered randomization envelope file to obtain the participant’s group assignment. The participant’s group allocation will be noted, and all opened randomization envelopes will be returned to the independent research coordinator to check for consistency in participant allocation. Typical of community-based RCTs with psychosocial interventions, blinding of research personnel, community partners, and participants to treatment allocation would not be possible owing to the nature of the intervention [32].

### Intervention

All study participants (n=24) will receive rent subsidies for 24 months, paid directly to the landlords by our community partners. Given the higher cost of rent in Toronto, youth living in Toronto will receive Can $500/month, whereas youth living in Hamilton and St Catharines will receive Can $400/month.

Participants in the intervention group (n=12) will be matched with an adult mentor recruited and screened by one of our community partners. Covenant House Toronto has an established mentorship program and will share their comprehensive mentor screening and training resources, which will act as a guide for all sites. Working with established community resources makes practical sense; not only will this facilitate colearning and capacity building between the research team and our community partners but delivering the mentorship intervention under *real-world* conditions will also provide important insights into scalability and sustainability [33,34]. The mentors will be encouraged to incorporate the key relationship-based components of natural mentors previously described (eg, a *coach* or *cheerleader* role) to help facilitate the connection of participants to larger social networks (including education and employment). All mentors will meet monthly with their mentees for 2 years and will be expected to be in contact with their mentee via phone or text message every week. If a mentor is unable to continue their role with more than 6 months remaining in the study, the participant will be matched with a new mentor.

**Figure 1:**
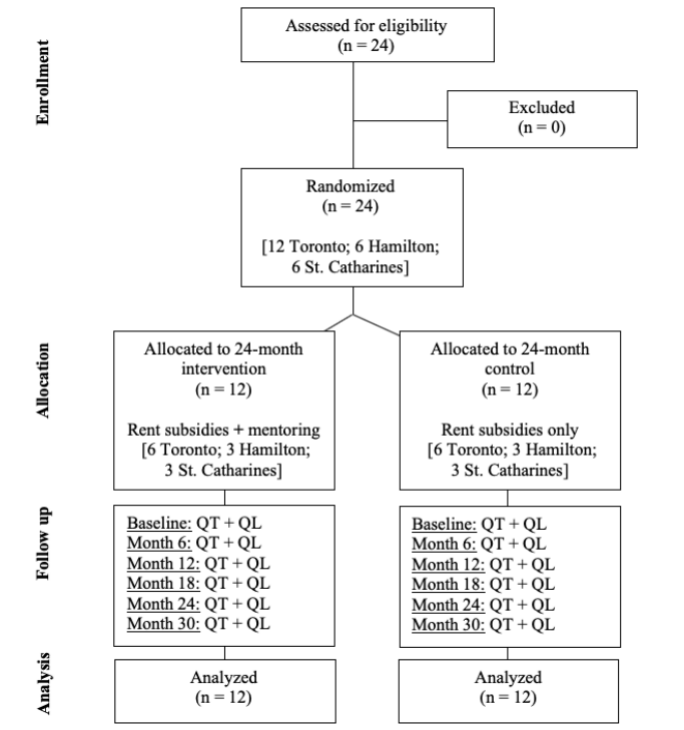
Consolidated standards of reporting trials’ diagram of ideal flow of participants through the study. The quantitative measures (QT) completed with all participants will comprise 6 standardized measures to assess community integration, self-esteem, social connectedness, hope, engulfment, and psychiatric symptoms. In addition, participants will complete 2 brief questionnaires pertaining to: (1) education (includes skills training), employment, and income and (2) perceived housing quality. The qualitative measures (QL) will comprise one-on-one semistructured interviews with the same 12 participants. The interview questions will explore the participant’s perspectives of the intervention and provide context to the quantitative responses.

Our community partners will match all participants with an outreach worker (already employed by each agency and considered standard of care) who will communicate regularly with the research team, help ensure the rent subsidies are being distributed appropriately, maintain an ongoing relationship with the study participants and mentors, and monitor for red flags in participants matched in mentor-mentee relationships (eg, mentee reluctant to meet with their mentor). Figure 1 summarizes the ideal flow of participants through the study.

### Study Outcomes

To fully apprehend the complex explanatory pathways of the intervention, we have aligned our key outcome variables (Table 1) with the Medical Research Council guidance on evaluating complex interventions and identified more than one primary outcome measure [35,36]. The primary outcome measures for this study are community integration (psychological and physical) and self-esteem at 18 months. Secondary outcomes include social connectedness, hope, and academic and vocational participation at 18 months. Exploratory outcomes include engulfment, psychiatric symptoms, income, perceived housing quality, and participant perspectives of intervention barriers and facilitators.

**Table 1:** Key outcome variables.

Variables		Instruments
**Primary outcomes**		
	Community integration (psychological and physical)	Community Integration Scale [37]
	Self-esteem	Rosenberg Self-Esteem Scale [38]
**Secondary outcomes**		
	Social connectedness	Social Connectedness Scale–Revised [39]
	Hope	Beck Hopelessness Scale [40]
	Enrollment in education (includes skills training)	Composite checklist
	Employment	Composite checklist
**Exploratory outcomes**		
	Income	Composite checklist
	Engulfment	Modified Engulfment Scale [41]
	Psychiatric symptoms	Modified Colorado Symptom Index [42]
	Perceived housing quality	Perceived Housing Quality Scale [43]
	Participant perspectives of barriers and facilitators	Individual semistructured interviews (youth) and focus groups (mentors) and composite checklist (mentor evaluation)

### Quantitative Data Collection

Participant demographic data will be collected at baseline. Quantitative questionnaires (Table 2) will be completed at 6 points in time over the course of 30 months: baseline, month 6, month 12, month 18, month 24, and month 30. Instruments previously utilized in research with young people who have experienced homelessness [14,44] were chosen so that meaningful comparisons can be made across studies [36]. Participants will be paid an honorarium of Can $20 at each of the 6 quantitative data collection points.

**Table 2:** Quantitative data collection.

Instrument	Psychometric information
Beck Hopelessness Scale [40]	This 20-item scale measures motivation, expectations, and feelings about the future (internal consistency alpha=.93)
Community Integration Scale [37]	This 11-item scale measures behavioral (eg, participation in activities) and psychological (eg, sense of belonging) aspects of community integration. This scale was used extensively in the Chez Soi/At Home study [37], but psychometric properties have yet to be reported
Education, Employment, and Income Questionnaire	This 13-item questionnaire assesses education, employment, and income. We developed this questionnaire for the study
Mentor Evaluation Questionnaire	This 10-item questionnaire assesses mentor effectiveness. It will be completed at month 24 by those in the intervention group. We developed this questionnaire for the study in collaboration with our community partners
Modified Colorado Symptom Index [42]	This 14-item scale measures the presence and frequency of psychiatric symptoms experienced in the past month (internal consistency alpha=.90 to .92)
Modified Engulfment Scale [41]	This 30-item scale measures the degree to which an individual’s self-concept is defined by their experience of homelessness (internal consistency alpha=.91). We have adapted the scale for this study, substituting *experience of homelessness* for *illness*
Perceived Housing Quality [43]	This 7-item scale measures participants’ perception of housing choice and quality. This scale was used extensively in the At Home/Chez Soi study [37], but psychometric properties have yet to be reported. We have shortened it from 10 items (At Home/Chez Soi) to 7 relevant items
Rosenberg Self-Esteem Scale [38]	This 10-item scale measures global self-worth (internal consistency alpha=.77 to .88)
Social Connectedness Scale–Revised [39]	This 20-item scale measures belongingness—the degree to which an individual feels connected to others (internal consistency alpha=.92)

### Qualitative Data Generation

Qualitative measures are an important feature of this study and will comprise semistructured individual interviews with study participants and focus groups with mentors. A total of 12 participants (6 from Toronto, 3 from Hamilton, and 3 from St Catharines) will be purposively chosen to participate in 6 semistructured individual interviews, which will take place at baseline, month 6, month 12, month 18, month 24, and month 30. We will select 6 participants from the intervention group (rent subsidies plus mentorship) and 6 from the control group (rent subsidies only) for qualitative interviews. All mentors (n=12) will be invited to participate in 2 focus groups, which will take place at month 12 and month 24. The questions posed during the semistructured interviews and focus groups will be conversational and exploratory in nature with particular attention to understanding how mentoring and/or rent subsidies influence social integration outcomes for formerly homeless young people living in market rent housing. To get a better sense of each young person’s living situation and to minimize researcher-participant power imbalance [33], the individual interviews will take place in or nearby the young people’s homes. The individual interviews and focus groups will be audio recorded and transcribed verbatim. Given the emergent nature of qualitative inquiry, we expect the interview and focus group questions to evolve over time as key preliminary themes begin to surface [45,46]. Young people participating in semistructured interviews will be paid an honorarium of Can $30 at each interview.

### Data Analysis

One major critique of mixed methods RCTs is that, typically, there is no true integration (ie, *mixing*) of quantitative and qualitative findings at the level of analysis or interpretation [23]. Moreover, it is often unclear whether or how the quantitative and qualitative researchers have worked together to maximize the potential synergies between these different approaches [23]. With this in mind, our study team, comprising researchers with quantitative and qualitative expertise, worked together to develop this study protocol and anticipate meeting quarterly to discuss the emerging analysis and to explore (and follow up on) similarities or discrepancies between the quantitative and qualitative data to determine how the subjective experiences and statistical analysis compare.

#### Quantitative Analysis

All analyses will be performed using the intention-to-treat principle. Baseline characteristics of the intervention and control groups will be summarized using mean, standard deviation, median, and interquartile range for continuous variables and frequencies and proportions for categorical variables. Differences in group trajectories from baseline to 30 months follow-up will be explored using scatterplots and box plots. Group mean differences with 95% confidence intervals in continuous outcomes at 18 months (psychological community integration, self-esteem, social connectedness, hope, perceived housing quality, sense of engulfment, and psychiatric symptoms) will be estimated using analysis of covariance, including an indicator of intervention group and the baseline value of the outcome. We will perform regression diagnostics and will repeat analyses using the nonparametric Wilcoxon rank sum test if there are extreme outliers or influential observations. For the count outcome of physical community integration, groups will be compared at 18 months using graphical tools and the nonparametric Wilcoxon rank sum test. For binary outcomes at 18 months, such as sustained academic and vocational participation, and income above low income cut-off, differences in proportions with 95% confidence intervals will be estimated and tested using the chi-square or Fisher exact test. If there are high rates of attrition or missing interviews, we will consider performing multiple imputation.

#### Qualitative Analysis

In keeping with the emergent, iterative nature of research using a qualitative design [45,46], data analysis and interpretation will begin immediately after the first qualitative data generation session at baseline. To conduct a more nuanced analysis of the data, the transcriptionist will be instructed to note short responses, uncooperative tones, and literal silence [46,47]. Before each subsequent qualitative data generation session, members of the research team will conduct a preliminary data analysis, reading the interview transcripts multiple times, separating the data into coded segments, making analytic memos beside sections of the transcripts, identifying emerging themes, and comparing and contrasting these among respondents, and compiling new questions [21,45]. Those participating in the individual interviews and the focus groups will be asked for their perspectives on the emerging interpretations at each visit and these perspectives will play a key role in helping shape the data analysis and help ensure the trustworthiness of the data [21,48]. The Web-based application Dedoose will be utilized to assist with sorting and coding the qualitative data.

### Public Involvement

We worked closely with our community partners to design this study and amended the design based on their feedback. For example, we initially proposed a study design where only half the young people would receive rent subsidies with the other half receiving *treatment as usual*; however, we abandoned this idea after our community partners challenged the ethics of not providing rent subsidies to young people living a precarious existence and desperate for immediate, tangible support to help them remain in market rent housing. In addition, we have formed a community advisory board and are meeting on a semiannual basis. We are also in the process of developing a newsletter to disseminate emerging findings to our community partners.

### Ethics and Dissemination

All data collected will be kept in strict confidence. Although the participants’ names will appear on the consent forms, pseudonyms (created by the participants) will be used in place of their real names on all documents related to data generation, including the audio recordings and interview transcripts. All electronic data will be stored on a secure server at the MAP Centre for Urban Health Solutions at St Michael’s Hospital and will be accessible only to select members of the research team. The audio recordings from the individual interviews and focus groups will be deleted once the transcripts have been stored on the secure server and entered into the Dedoose application. Paper copies of the data (eg, consent forms and standardized quantitative measures) will be stored in a locked filing cabinet at the MAP Centre for Urban Health Solutions—an area accessible only to those with electronic key access. All paper and electronic files will be retained for a period of up to 5 years from study closure.

In keeping with our CBPAR methodology, we are committed to disseminating evidence *with* our community partners to build community capacity and improve the lives of the young people participating in this study [24,27]. With an emphasis on *actionable* data [24], we anticipate disseminating our findings broadly to both academic and community-based audiences in a variety of formats ranging from scientific journal papers to oral presentations. Deidentified participant data will be available from the lead author upon request once the study is completed.

## Results

Ethical approval for this study (protocol version January 13, 2019) was obtained in November 2018 from the Providence, St Joseph’s, and St Michael’s Research Ethics Board (REB; number 18-251). Any amendments to the study protocol will be reviewed by our community partners and approved by the Providence St Joseph’s and St Michael’s Healthcare REB before implementation. Enrollment took place from April 2019 to September 2019. We have enrolled 24 young people in the study. Preliminary analysis of the baseline quantitative and qualitative data is underway.

## Discussion

This pilot RCT will be the first to test the impact of economic and social support on meaningful social integration for formerly homeless young people living in market rent housing. We believe the mixed methods design will illuminate important contextual factors that must be considered if the intervention is to be scaled up and replicated elsewhere. Importantly, the CBPAR framework will incorporate the perspectives of the community, including formerly homeless young people, who are in the best position to determine what might work best in the context of their lives.

Young people recruited for this pilot study will be a small sample of youth connected to urban-based social service providers in the province of Ontario, Canada. Thus, the findings may not be generalizable to formerly homeless young people living in other contexts and/or not connected to social service agencies.

## Abbreviations

CBPAR: community-based participatory action research
REB: Research Ethics Board
RCT: randomized controlled trial
